# Feedback GABA signaling suppresses white spot syndrome virus infection via mTORC1-mediated autophagy inhibition in shrimp

**DOI:** 10.1128/mbio.01288-26

**Published:** 2026-07-13

**Authors:** Jie Gao, Ye-Jia Song, Xin-Shuang Zhang, Dong-Dong Han, Dongwei Kang, Yingjie Li, Xian-Wei Wang

**Affiliations:** 1 School of Life Sciences, Shandong University 520252, Qingdao, China; 2 Department of Medicinal Chemistry, School of Pharmaceutical Sciences, Cheeloo College of Medicine, Shandong University 66555, Jinan, China; 3 State Key Laboratory of Microbial Technology, Shandong University 520252, Qingdao, China; Tsinghua University, Beijing, China

**Keywords:** white spot syndrome virus, autophagy, gamma-aminobutyric acid, shrimp

## Abstract

Gamma-aminobutyric acid (GABA) is a neurotransmitter whose role in virus-host interactions remains poorly defined. Here, we show that GABA restricts white spot syndrome virus (WSSV) infection by suppressing virus-induced autophagy in kuruma shrimp *Marsupenaeus japonicus*. Mechanistically, GABA promotes extracellular calcium influx in a GABA type A receptor (GABA_A_R)-dependent manner, leading to activation of mTORC1 signaling. This pathway suppresses virus-induced autophagy, thereby interfering with the early stage of WSSV infection. Consistent with this model, pharmacological inhibition of autophagy recapitulated the antiviral effect of GABA, whereas autophagy activation counteracted GABA-mediated restriction of infection. We further found that autophagy inhibition caused accumulation of the autophagy adaptor P62, which was associated with reduced membrane localization of GABA_A_R and attenuation of GABA signaling. These results suggest a feedback mechanism that restrains excessive autophagy suppression. Together, these findings identify a GABA-GABA_A_R-Ca^2+^-mTORC1 signaling axis that suppresses virus-induced autophagy and thereby restricts WSSV infection.

**IMPORTANCE:**

White spot syndrome virus (WSSV) exploits host autophagy to promote infection, but the host signals that regulate this process remain poorly understood. Here, we show that gamma-aminobutyric acid, a classical neurotransmitter, restricts WSSV infection in kuruma shrimp by suppressing virus-induced autophagy through a GABA_A_R-Ca^2+^-mTORC1 signaling pathway. We further identify a P62-mediated feedback mechanism that restrains this response. These findings define a host signaling mechanism that controls a proviral autophagy-dependent step in WSSV infection and reveal that neurotransmitter signaling can contribute to host control of viral infection in an invertebrate.

## INTRODUCTION

White spot syndrome virus (WSSV) is a devastating enveloped DNA virus of shrimp aquaculture and relies extensively on host cellular processes to complete its infection cycle. Autophagy is a conserved catabolic pathway that maintains cellular homeostasis by delivering cytoplasmic components to lysosomes for degradation and recycling, particularly under conditions of nutrient limitation, organelle damage, or infection-induced stress (1, 2). During viral infection, autophagy can play opposing roles, depending on the viral and host context (3). Autophagy can target viral components or virus-containing compartments for lysosomal degradation. Many viruses, however, subvert autophagy or autophagy-related processes to support entry, replication, assembly, egress, or metabolic adaptation (4, 5). Autophagy therefore directly influences the outcome of viral infection. In crustaceans, increasing evidence indicates that autophagy contributes to WSSV infection. Our previous work further showed that lipid autophagy promotes WSSV infection by supporting host lipid utilization, linking autophagy to the metabolic requirements of WSSV infection in shrimp (6). It remains unclear whether host signaling pathways can modulate this proviral autophagy and limit WSSV infection.

Gamma-aminobutyric acid (GABA) is best known as an inhibitory neurotransmitter in the nervous system, where it is synthesized from glutamate by glutamate decarboxylase and mediates diverse physiological functions through GABA receptors (7–9). GABA also functions outside the nervous system. Recent studies in vertebrates have shown that GABA is produced by and acts on non-neuronal cells, including immune cells, and regulates inflammatory and immune responses (10–12). For example, GABA signaling has been linked to the control of inflammasome activation, macrophage inflammatory activity, and lymphocyte-associated immune regulation (13, 14). These studies suggest that GABA functions not only as a neurotransmitter but also as an immune-regulatory signal. The effect of GABA on infection varies across systems. In some systems, GABA signaling enhances host defense or alleviates infection-associated pathology, whereas in others, it promotes pathogen infection or replication. In mice, GABA mitigates influenza-associated pathology and promotes viral clearance (15). By contrast, in mice, GABA has also been reported to enhance hepatitis B virus replication (16). In mosquitoes, GABAergic signaling facilitates arbovirus infection by modulating innate immune responses (17). These findings show that GABA can either promote or restrict infection, but how it does so remains unclear.

GABA has been linked to the control of autophagy in multiple systems. Previous studies indicate that GABA can modulate autophagy in a context-dependent manner, including inhibition of selective autophagy in yeast and mice and enhancement of autophagy in rat mesenteric lymph node cells and murine macrophages (18–20). Given that WSSV exploits autophagy to promote infection in shrimp, this connection raised the possibility that GABA may affect WSSV infection by modulating virus-induced autophagy. Here, we found that GABA suppresses virus-induced autophagy and restricts WSSV infection. Mechanistically, GABA promotes extracellular calcium influx in a GABA type A receptor (GABA_A_R)-dependent manner, leading to activation of mTORC1 signaling and consequent suppression of virus-induced autophagy. We further found that inhibition of autophagy caused P62 accumulation, which in turn imposes feedback restraint on this signaling pathway. Together, these findings identify a host pathway through which neurotransmitter signaling suppresses virus-induced autophagy and restricts WSSV infection.

## RESULTS

### GABA inhibits viral infection in shrimp

**Fig 1:**
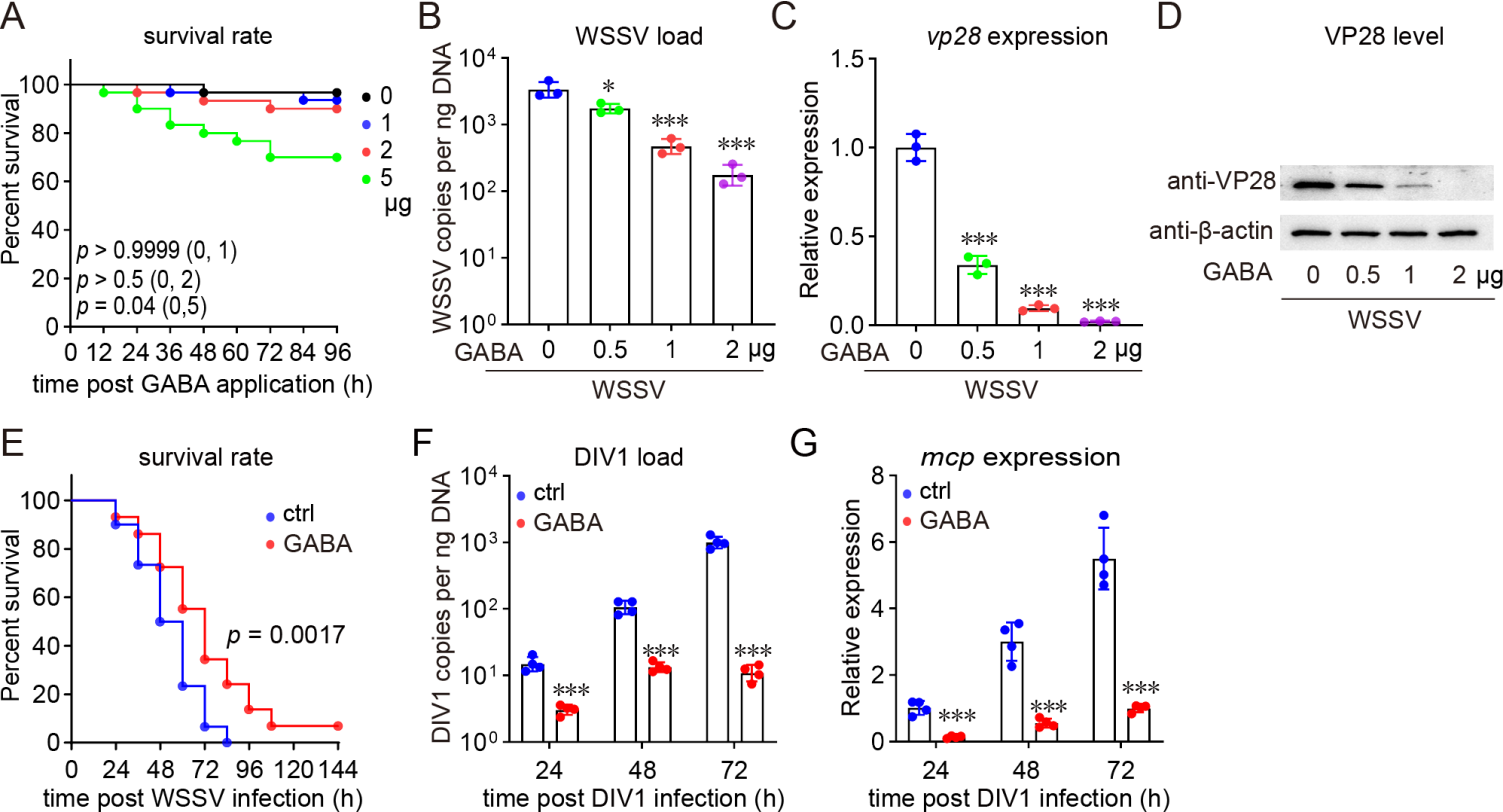
GABA protects shrimp from WSSV and DIV1 infection. (**A**) Effect of GABA on shrimp survival rate. Shrimp were injected with 1, 2, or 5 μg GABA per shrimp. PBS-injected shrimp served as controls. Survival rates were recorded every 12 h for 96 h (*n* = 30). (**B–D**) GABA suppresses WSSV infection in shrimp. Shrimp were infected with WSSV (5 × 10^5^ copies) at 2 h post-GABA injection (2 μg). Viral load (**B**) and *vp28* transcription (**C**) were quantified by qPCR, while VP28 protein levels (**D**) were analyzed by Western blotting at 48 h post-infection. (**E**) GABA enhances survival of WSSV-infected shrimp. WSSV (1 × 10^6^ copies) was injected at 2 h post-GABA treatment (2 μg). Survival was monitored every 12 h for 144 h (*n* = 30). (**F and G**) GABA inhibits DIV1 infection in shrimp. DIV1 (5 × 10^5^ copies) was injected at 2 h post-GABA treatment. DIV1 load (**F**) and *mcp* transcription (**G**) were quantified by qPCR. All bar chart data are presented as mean ± SD of three or four replicates. ***, *P* < 0.001; *, 0.01 < *P* < 0.05, as determined using Student’s *t*-test*.* The survival data are representative of three biological replicates and analyzed by log-rank (Mantel-Cox) test. The Western blotting images are representative of three independent replicates.

To determine an appropriate dose of GABA for subsequent experiments, we first evaluated the effects of different doses on shrimp survival. Treatment with 1 or 2 μg GABA did not significantly affect survival, whereas 5 μg GABA reduced shrimp survival by approximately 25% compared with that in the control group (Fig. 1A). These results indicate that a GABA dose of 2 μg or less does not significantly affect the survival of shrimp. We next examined the effect of GABA on WSSV infection in shrimp. GABA treatment significantly reduced viral load (Fig. 1B). A similar inhibitory effect was observed at both the transcript and protein levels of the major WSSV structural protein VP28 (Fig. 1C and D). In addition, GABA treatment significantly improved shrimp survival after WSSV infection (Fig. 1E). These results indicate that GABA protects shrimp from WSSV infection. GABA also inhibited infection by another shrimp virus, decapod iridescent virus 1 (DIV1) (Fig. 1F and G). These results suggest that GABA contributes to antiviral defense in shrimp.

### GABA inhibits WSSV infection by suppressing virus-induced autophagy

**Fig 2:**
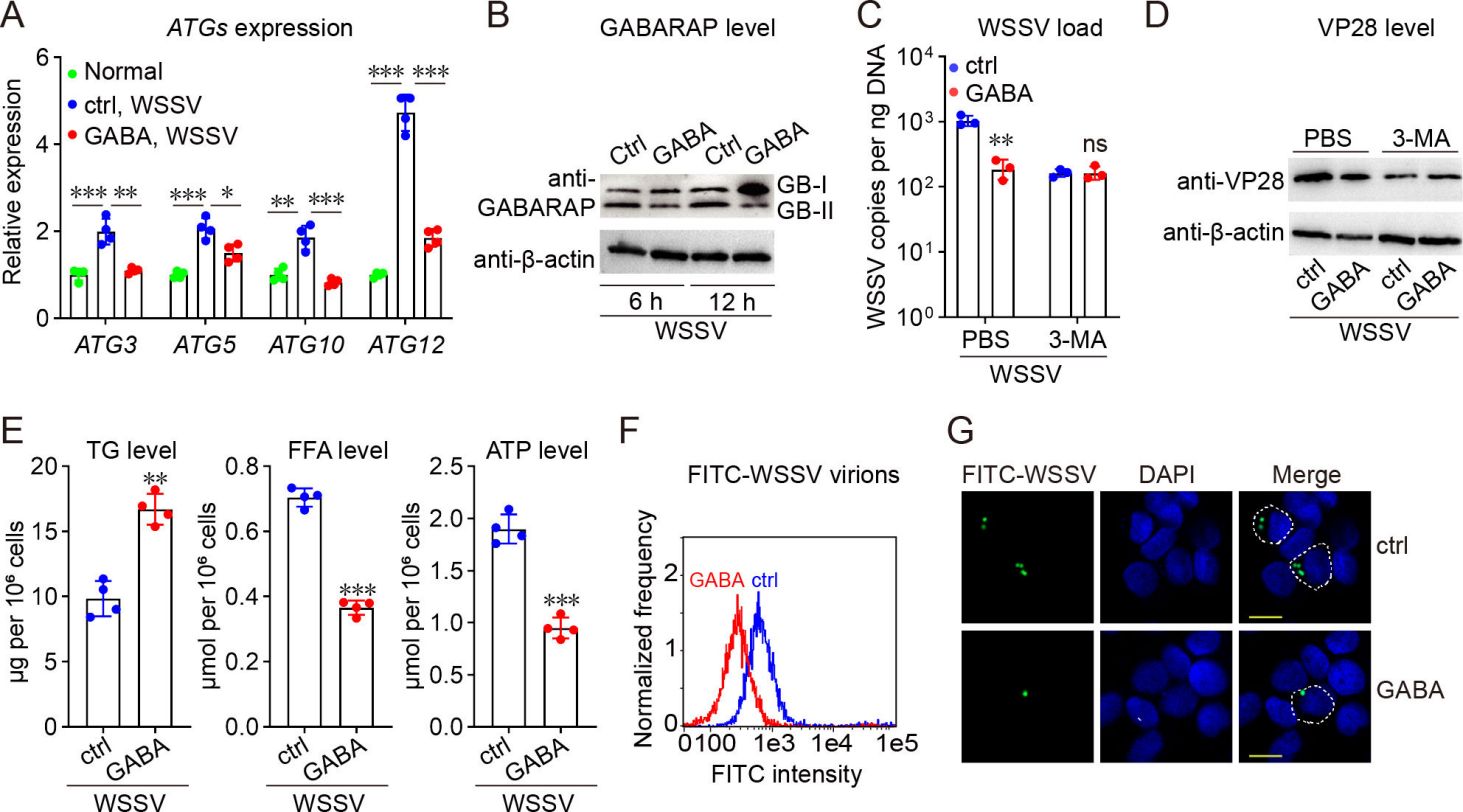
GABA inhibits viral infection by regulating autophagy. (**A**) Expression of autophagy-related genes after GABA treatment. Shrimp were infected with WSSV (5 × 10^5^ copies) 2 h after GABA application (2 μg). Hemocytes’ total RNA was collected 6 h post-infection. Relative expression of autophagy-related genes was analyzed by qPCR. (**B**) GABA suppresses WSSV-induced autophagic activity. Shrimp were infected with WSSV (5 × 10^5^ copies) 2 h after GABA treatment (2 μg). Total protein was extracted from hemocytes, and GABARAP-II (GB-II) levels were analyzed by Western blotting. (**C and D**) Role of 3-MA in GABA-mediated inhibition of WSSV infection. Shrimp were co-treated with GABA (2 μg) and 3-MA 2 h before WSSV infection (5 × 10^5^ copies). Hemocytes were collected 48 h post-infection. WSSV load (**C**) and VP28 levels (**D**) were measured by qPCR and Western blotting, respectively. (**E**) GABA modulates WSSV-induced lipid mobilization. Shrimp were infected with WSSV (5 × 10^5^ copies) 2 h after GABA treatment (2 μg). Hemocytes were collected 12 h post-infection. Triglyceride (TG), free fatty acid (FFA), and ATP levels were determined using commercial kits. (**F and G**) GABA suppresses an early stage of WSSV infection. Shrimp were infected with FITC-labeled WSSV (1 × 10^6^ copies) 2 h after GABA application (2 μg). Hemocytes were collected 2 h post-infection. WSSV signal was assayed by flow cytometry (**F**) and observed by confocal microscopy (**G**). All bar chart data are presented as mean ± SD of three or four replicates. ***, *P* < 0.001; **, 0.001 < *P* < 0.01; *, 0.01 < *P* < 0.05; ns, no significant difference, as determined using Student’s *t*-test. The Western blotting images are representative of three independent replicates.

Previous studies have shown that GABA can modulate autophagy in a context-dependent manner, impairing selective autophagy in yeast and mice while enhancing autophagy in rat mesenteric lymph node cells and murine macrophages *in vitro* (18–20). Our previous work showed that WSSV hijacks shrimp autophagy to facilitate viral entry and replication (6). Based on these observations, we hypothesized that GABA may exert an antiviral effect by regulating autophagy. To determine whether GABA affects autophagic activity during WSSV infection, we first measured the transcription of autophagy-related genes. WSSV infection significantly increased the transcript levels of ATG3, ATG5, ATG10, and ATG12, although the magnitude of induction varied among these genes, whereas GABA treatment markedly suppressed this induction (Fig. 2A). We next examined the level of GABARAP-II (GB-II), a marker of autophagic activity. GABA treatment inhibited the WSSV-induced increase in GB-II levels (Fig. 2B). These results indicate that GABA suppresses WSSV-induced autophagic activity.

We next investigated whether GABA inhibits WSSV infection by suppressing autophagy. Treatment with either GABA or the autophagy inhibitor 3-methyladenine (3-MA) reduced viral infection, and their effects were not additive (Fig. 2C and D), suggesting that GABA inhibits WSSV infection by acting on an autophagy-dependent process. Our previous studies have shown that WSSV exploits autophagy to degrade triglycerides (TGs), thereby generating free fatty acids (FFAs) and ATP to facilitate viral entry into cells (6). We therefore examined whether GABA affects these infection-associated metabolic changes. GABA treatment increased TG levels and reduced FFA and ATP levels in WSSV-infected shrimp (Fig. 2E). We further examined the early WSSV signal in hemocytes using fluorescein isothiocyanate (FITC)-labeled WSSV. Flow cytometry showed that GABA treatment significantly reduced FITC fluorescence intensity in hemocytes (Fig. 2F). Confocal microscopy likewise showed reduced virus-associated fluorescence in hemocytes from the GABA-treated group compared with that in the control group (Fig. 2G). Together, these results indicate that GABA inhibits WSSV infection by suppressing virus-induced autophagy and the associated metabolic changes.

### GABA activates mTORC1 signaling to suppress autophagy and inhibit WSSV infection

**Fig 3:**
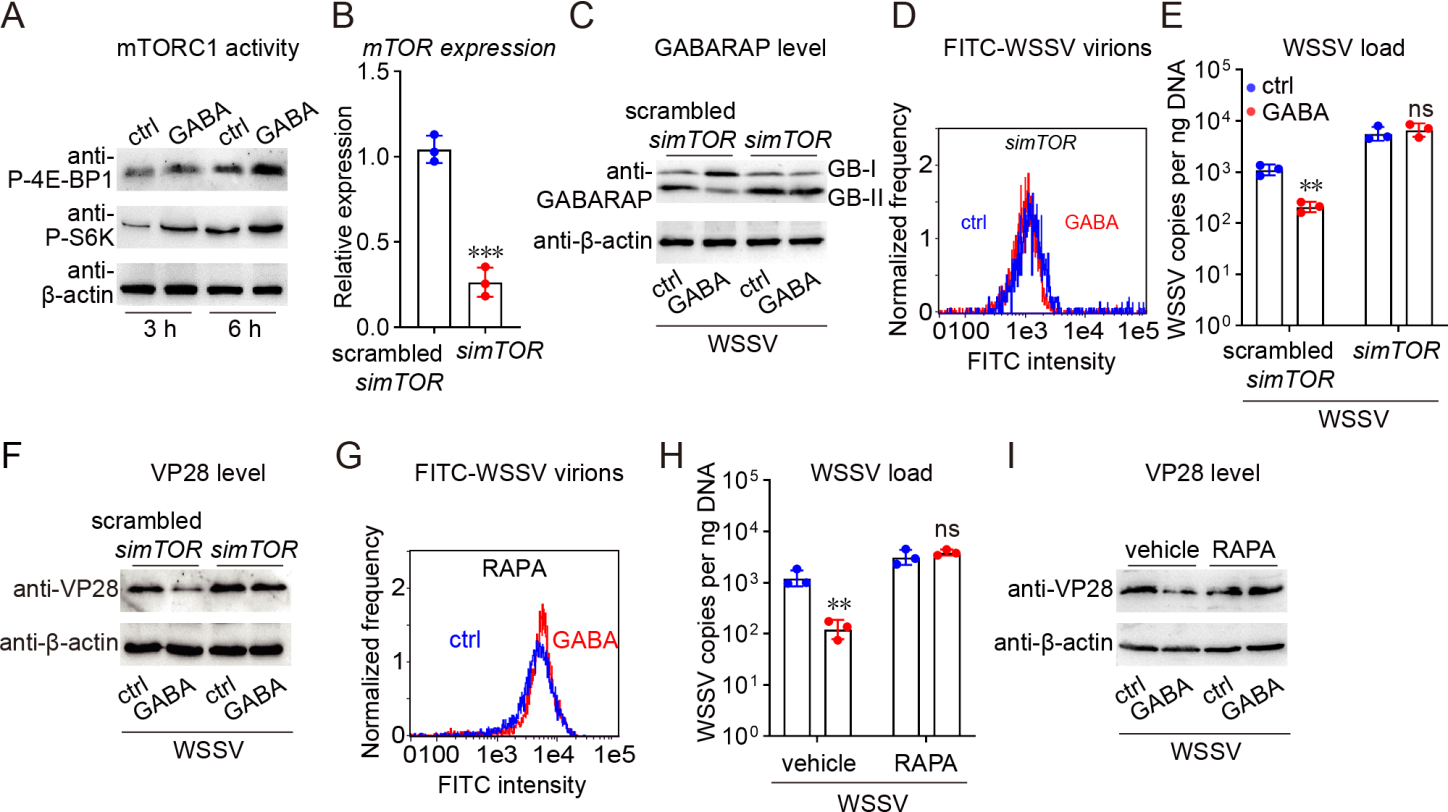
GABA inhibits autophagy by activating mTORC1. (**A**) GABA activates mTOR signaling. Hemocytes were collected at 3 and 6 h post-GABA treatment (2 μg). Phosphorylation of mTOR downstream targets (4E-BP1 and S6K) was analyzed by Western blotting. (**B**) Validation of mTOR knockdown. Shrimp were injected with *simTOR* (30 μg). *mTOR* transcript levels were quantified by qPCR 24 h post-injection. (**C**) mTOR knockdown abolishes GABA-mediated autophagy suppression. Shrimp were subjected to *simTOR* (30 μg) injection for 24 h prior to GABA treatment. GABARAP-II levels were assessed by Western blotting 12 h post-GABA administration. (**D**) mTOR is required for the inhibitory effect of GABA on the early stage of WSSV infection. Shrimp were injected with *simTOR* (30 μg) for 24 h, followed by GABA treatment (2 μg) for an additional 2 h, and then injected with FITC-labeled WSSV (1 × 10^6^ copies). Hemocytes were analyzed by flow cytometry 2 h post-injection. (**E and F**) GABA requires mTOR to inhibit WSSV replication. Shrimp were injected with *simTOR* (30 μg) for 24 h, followed by GABA treatment (2 μg) for an additional 2 h, and then infected with WSSV (5 × 10^5^ copies). Viral load (**E**) and VP28 levels (**F**) were detected by qPCR and Western blotting 48 h post-infection. (**G**) RAPA reverses the inhibitory effect of GABA during the early stage of WSSV infection. Shrimp were pretreated with RAPA (20 μg) for 2 h, followed by GABA treatment (2 μg) for an additional 2 h, and then injected with FITC-labeled WSSV (1 × 10^6^ copies). Hemocytes were analyzed by flow cytometry 2 h post-injection. (**H and I**) RAPA abrogates GABA-mediated suppression of WSSV infection. Shrimp were pretreated with RAPA (20 μg) for 2 h, and PBS containing 10% DMSO served as the corresponding vehicle control. Shrimp were then treated with GABA (2 μg) for an additional 2 h and subsequently infected with WSSV (5 × 10^5^ copies). Viral load (**H**) and VP28 levels (**I**) were evaluated by qPCR and Western blotting 48 h post-infection. All bar chart data are presented as mean ± SD of three or four replicates. ***, *P* < 0.001; **, 0.001 < *P* < 0.01; ns, no significant difference as determined using Student’s *t*-test. The Western blotting images are representative of three independent replicates.

We next explored the mechanism by which GABA suppresses host autophagy during WSSV infection. Previous studies in yeast and mice have shown that elevated GABA can impair selective autophagy through an mTOR-sensitive mechanism, and mTOR is a central regulator of autophagy (18, 21). We therefore examined whether GABA suppresses host autophagy during WSSV infection through mTORC1 activation. To determine whether mTORC1 signaling is involved in GABA-mediated suppression of autophagy, we first examined the phosphorylation of the mTORC1 downstream targets 4E-BP1 and S6K. GABA treatment significantly increased the phosphorylation levels of both 4E-BP1 and S6K compared with those in the control group (Fig. 3A), indicating that GABA activates mTORC1 signaling in shrimp.

We next examined whether mTORC1 activation is required for the effects of GABA on autophagy and WSSV infection. Knockdown of mTOR by small interfering RNA (siRNA) (Fig. 3B) markedly impaired the ability of GABA to suppress autophagy and inhibit the early stage of WSSV infection in shrimp (Fig. 3C and D). Consistently, the inhibitory effects of GABA on viral load and VP28 expression were also abolished by mTOR knockdown (Fig. 3E and F). To further validate these results, shrimp were treated with the mTORC1 inhibitor rapamycin (RAPA). RAPA treatment likewise eliminated the inhibitory effect of GABA on the early stage of WSSV infection (Fig. 3G), viral load, and VP28 expression (Fig. 3H and I). Together, these results indicate that GABA inhibits WSSV infection by activating mTORC1 signaling to suppress autophagy.

### GABA activates mTORC1 via calcium influx-dependent signaling

**Fig 4:**
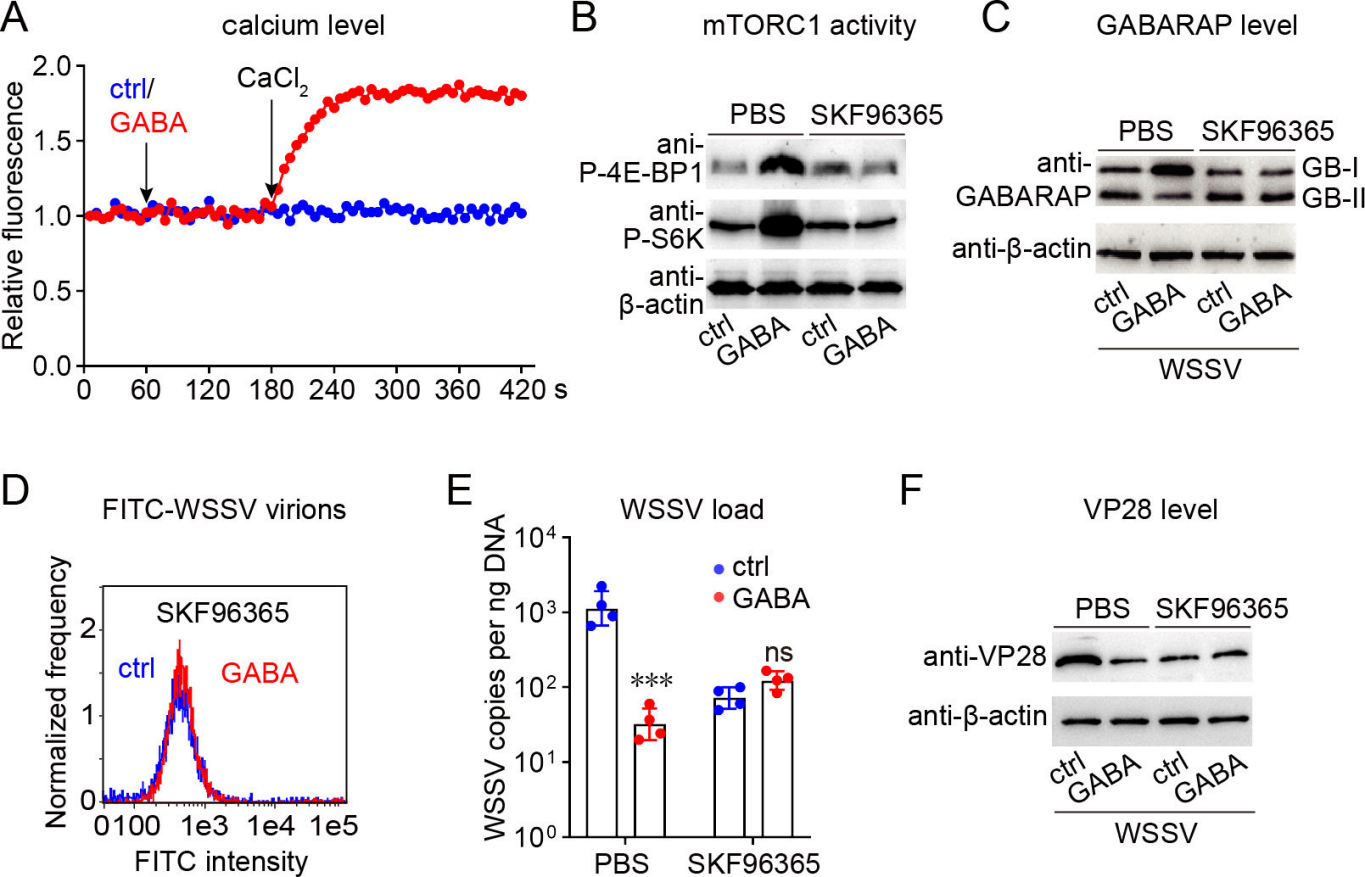
GABA activates mTORC1 through calcium-mediated signaling. (**A**) GABA induces calcium influx in hemocytes. Hemocytes were isolated from shrimp for *in vitro* culture, loaded with a calcium ion indicator for 30 min, and imaged via laser confocal microscopy. Images were captured every 6 s. PBS/GABA and CaCl_2_ (1 mM) were added at 1 and 3 min, respectively. Fluorescence signals were quantified using ImageJ. (**B**) Calcium influx is required for GABA-mediated mTOR activation. Shrimp were pretreated with the calcium channel blocker SKF96365 (5 μg) 2 h prior to GABA application. Hemocyte proteins were collected 6 h post-GABA treatment, and phosphorylation of mTOR targets (4E-BP1 and S6K) was analyzed by Western blotting. (**C**) GABA suppresses autophagy via the influx of extracellular calcium ions. Shrimp were pretreated with SKF96365 (5 μg) for 2 h, followed by GABA treatment (2 μg) for an additional 2 h, and then infected with WSSV (5 × 10^5^ copies). GABARAP-II levels were assessed by Western blotting 12 h post-infection. (**D**) SKF96365 reverses the inhibitory effect of GABA during the early stage of WSSV infection. Shrimp were pretreated with SKF96365 (5 μg) for 2 h, followed by GABA treatment (2 μg) for an additional 2 h, and then injected with FITC-labeled WSSV (1 × 10^6^ copies). Hemocytes were analyzed 2 h post-injection via flow cytometry. (**E and F**) Calcium signaling mediates GABA’s inhibition of WSSV infection. Shrimp were pretreated with SKF96365 (5 μg), followed by GABA (2 μg) 2 h later. WSSV (5 × 10⁵ copies) was administered 2 h post-GABA treatment. Viral load (**E**) and VP28 levels (**F**) were detected by qPCR and Western blotting 48 h post-infection. All bar chart data are presented as mean ± SD of three or four replicates. ***, *P* < 0.001; ns, no significant difference, as determined using Student’s *t*-test. The Western blotting images are representative of three independent replicates.

We next investigated how GABA activates mTORC1. Previous studies in human brain microvascular endothelial cells have shown that GABA can elevate intracellular calcium levels (22). In addition, in mammalian cells, extracellular calcium influx has been reported to promote calcium-dependent binding of calmodulin to hVps34, thereby triggering mTORC1 activation (23). These observations suggested a potential mechanism in which GABA promotes extracellular Ca^2+^ influx, increases cytosolic Ca^2+^ levels, and thereby activates mTORC1 signaling. To test this possibility, we first measured intracellular Ca^2+^ levels. GABA treatment significantly increased Ca^2+^ fluorescence intensity compared with that in the control group (Fig. 4A), indicating that GABA elevates intracellular Ca^2+^ levels.

We next examined whether Ca^2+^ influx is required for GABA-induced mTOR activation. Treatment with the Ca^2+^ channel blocker SKF96365 abolished the ability of GABA to increase the phosphorylation levels of 4E-BP1 and S6K (Fig. 4B). SKF96365 also eliminated the inhibitory effect of GABA on GB-II levels (Fig. 4C), indicating that Ca^2+^ influx is required for GABA-mediated suppression of autophagic activity. After WSSV infection, SKF96365 further abolished the inhibitory effects of GABA on the early stage of WSSV infection, viral load, and VP28 expression (Fig. 4D through F). Together, these results indicate that GABA activates mTORC1 signaling through a Ca^2+^ influx-dependent mechanism.

### GABA_A_R mediates antiviral autophagy suppression through the Ca^2+^-mTORC1 axis

**Fig 5:**
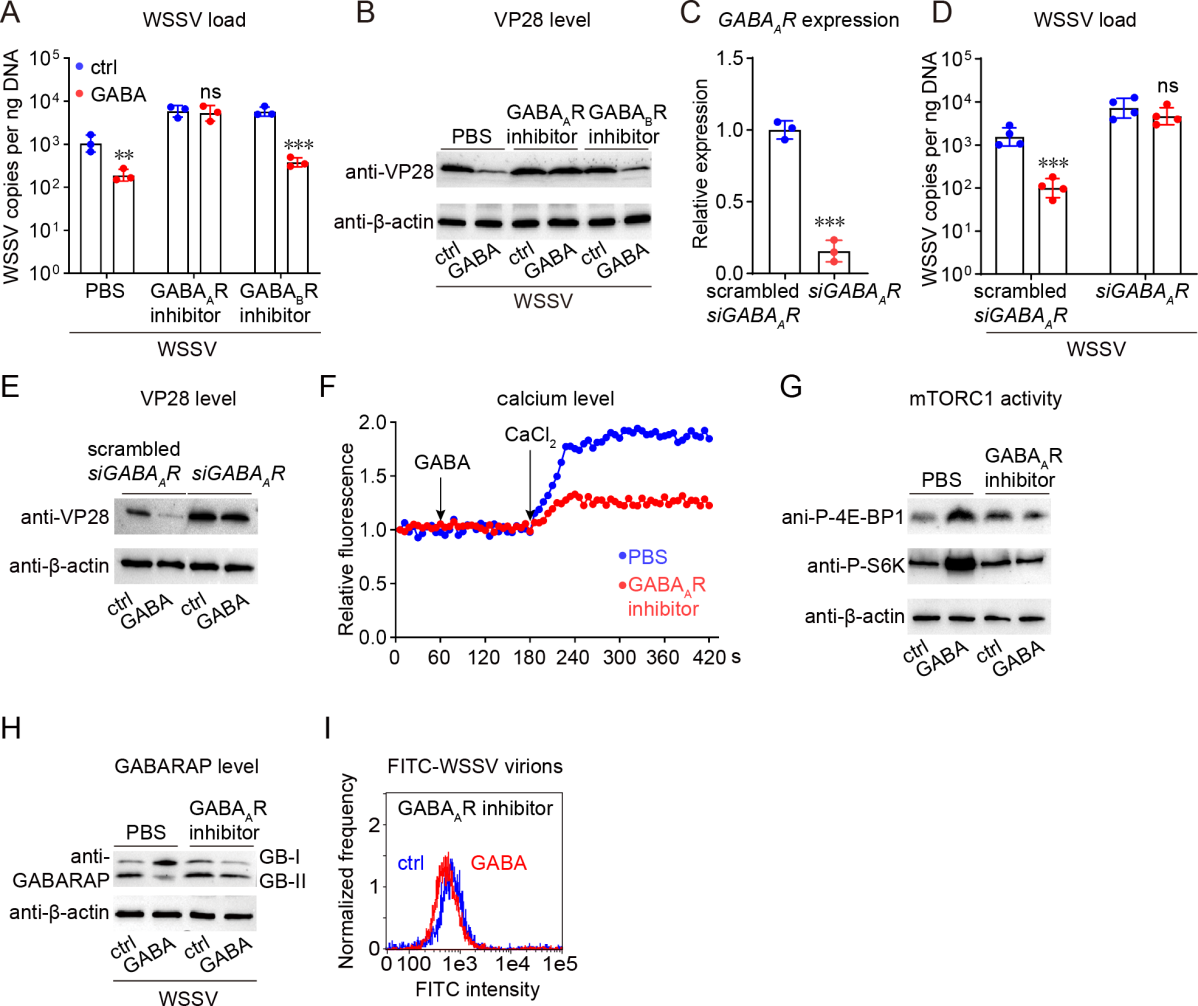
GABA_A_R mediates the antiviral effect of GABA. (**A and B**) Role of GABA_A_R and GABA_B_R inhibitors in GABA-mediated regulation of WSSV load. Shrimp were pretreated with GABA_A_R inhibitor (5 μg) and GABA_B_R inhibitor (2 μg) for 2 h, after which GABA (2 μg) was injected. WSSV infection (5 × 10^5^ copies) was performed 2 h post-GABA treatment. Hemocytes were collected 48 h post-infection. WSSV load (**A**) and VP28 levels (**B**) were analyzed by qPCR and Western blotting, respectively. (**C**) GABA_A_R knockdown efficiency. Shrimp were injected with *siGABA_A_R* (30 μg). *GABA_A_R* transcription was analyzed by qPCR 24 h post-injection. (**D and E**) Knockdown of the GABA_A_R impairs the antiviral effect of GABA. After GABA_A_R silencing, shrimp were treated with GABA (2 μg) for 2 h and then infected with WSSV (5 × 10^5^ copies). Viral load (**D**) and VP28 levels (**E**) were detected by qPCR and Western blotting 48 h post-infection. (**F**) Calcium influx assay in hemocytes. Hemocytes were extracted from shrimp for *in vitro* culture, treated with GABA_A_R inhibitor (5 μg) for 2 h, loaded with a calcium ion indicator for 30 min, and observed under a confocal microscope. Images were captured every 6 s. GABA (20 μM) and CaCl_2_ (1 mM) were added at 1 and 3 min, respectively. Fluorescence intensity was quantified using ImageJ software. (**G**) GABA_A_R inhibitor blocks GABA-induced mTOR activation. Shrimp were pretreated with GABA_A_R inhibitor (5 μg) for 2 h, followed by GABA administration (2 μg). Hemocytes were collected 6 h post-treatment, and phosphorylation levels of 4E-BP1 and S6K were detected by Western blotting. (**H**) GABA_A_R inhibitor reverses GABA-mediated autophagy suppression. Shrimp pretreated with GABA_A_R inhibitor were injected with GABA (2 μg). GABARAP-II levels in hemocytes were measured by Western blotting 12 h post-injection. (**I**) GABA_A_R inhibitor reverses the inhibitory effect of GABA during the early stage of WSSV infection. Shrimp were treated with GABA_A_R inhibitor (5 μg) for 2 h, after which GABA (2 μg) was injected. FITC-labeled WSSV infection (1 × 10^6^ copies) was performed 2 h post-GABA treatment. Hemocytes were analyzed 2 h post-injection via flow cytometry. All bar chart data are presented as mean ± SD of three or four replicates. ***, *P* < 0.001; **, 0.001 < *P* < 0.01; ns, no significant difference, as determined using Student’s *t*-test. The Western blotting images are representative of three independent replicates.

GABA generally exerts its biological functions through either the ionotropic type A receptor (GABA_A_R) or the metabotropic type B receptor (GABA_B_R) (22). To determine which receptor mediates the antiviral effect of GABA in shrimp, we treated shrimp with inhibitors of GABA_A_R or GABA_B_R and then assessed the effect of GABA on WSSV infection. Inhibition of GABA_A_R abolished the antiviral effect of GABA, whereas inhibition of GABA_B_R had no detectable effect (Fig. 5A and B). These results indicate that GABA_A_R, but not GABA_B_R, mediates the antiviral effect of GABA in shrimp. To further determine the role of GABA_A_R in the antiviral effect of GABA, we used RNA interference (RNAi) to reduce the expression of GABA_A_R in shrimp hemocytes (Fig. 5C). After receptor knockdown, the antiviral effect of GABA against WSSV infection was markedly weakened (Fig. 5D and E), supporting the involvement of hemocyte-expressed GABA_A_R-related signaling in the antiviral action of GABA.

We next examined whether GABA_A_R is required for GABA-induced Ca^2+^ elevation and subsequent mTORC1 activation. Treatment with the GABA_A_R inhibitor significantly reduced the ability of GABA to induce intracellular Ca^2+^ elevation in shrimp hemocytes (Fig. 5F). Consistently, GABA no longer increased the phosphorylation levels of 4E-BP1 and S6K after GABA_A_R inhibition (Fig. 5G), and its inhibitory effect on GB-II was also lost (Fig. 5H). In addition, GABA no longer inhibited the early stage of WSSV infection when GABA_A_R was inhibited (Fig. 5I). Together, these results indicate that the ability of GABA to activate mTORC1 signaling, suppress autophagy, and inhibit WSSV infection depends on GABA_A_R.

### P62 mediates negative feedback on GABA-induced autophagy suppression

**Fig 6:**
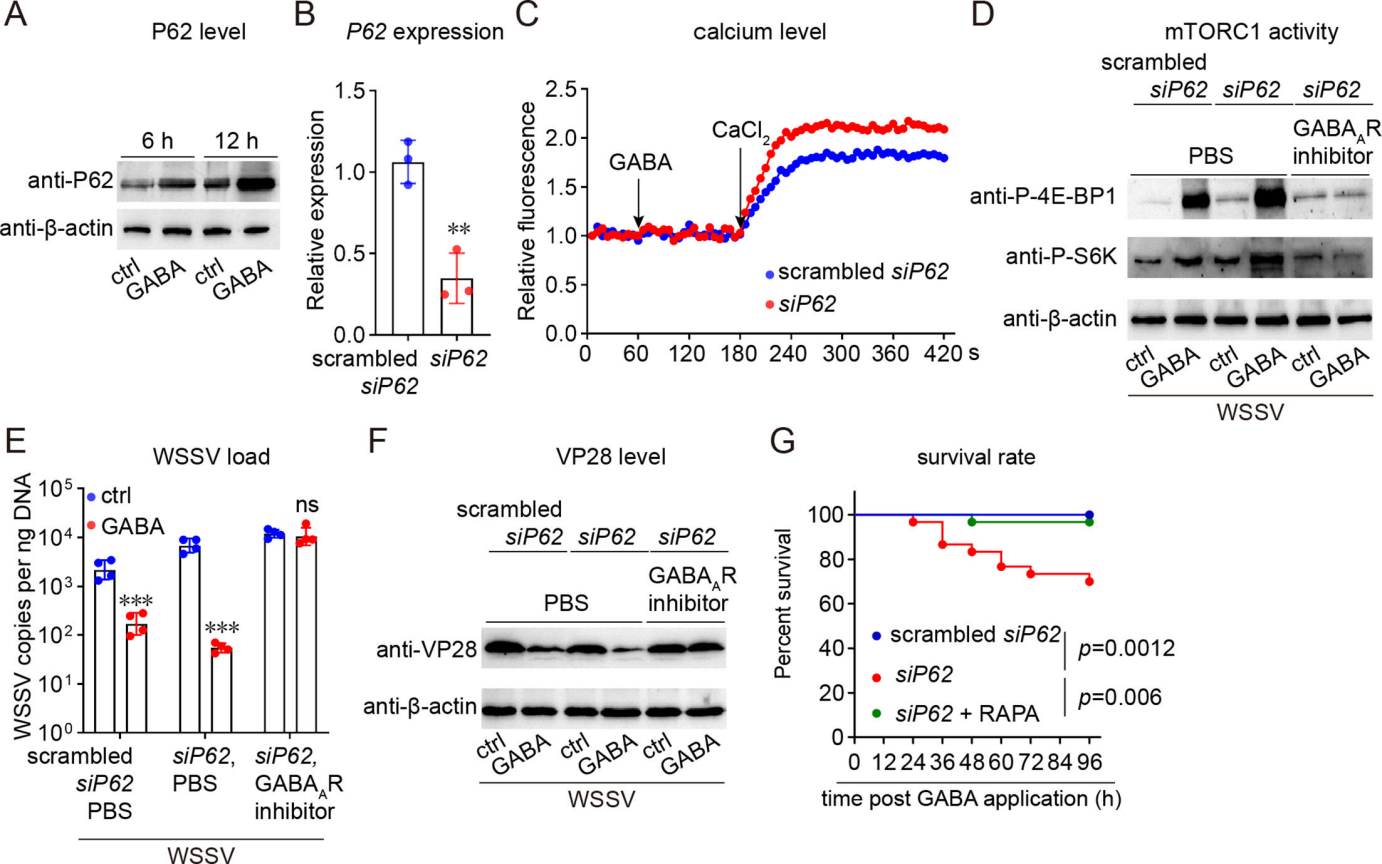
P62 negative feedback regulates GABA function. (**A**) GABA increases P62 protein levels. P62 levels were detected by Western blotting 6 and 12 h post-GABA treatment (2 μg). (**B**) P62-knockdown efficiency. Shrimp were injected with *siP62* (30 μg). *P62* transcription was analyzed by qPCR 24 h post-injection. (**C**) Calcium signaling in P62-knockdown hemocytes. Shrimp were injected with *siP62* (30 μg). Hemocytes were extracted from shrimp for *in vitro* culture 24 h after *siP62* injection. Prior to imaging, hemocytes were loaded with a calcium ion indicator for 30 min and observed under a confocal microscope. Images were captured every 6 s. GABA (20 μM) and CaCl_2_ (1 mM) were added at 1 and 3 min, respectively. Fluorescence intensity was quantified using ImageJ software. (**D**) P62 knockdown enhances GABA-induced mTORC1 activation, and this effect is abolished by GABA_A_R inhibition. After P62 silencing, shrimp were treated with GABA in the presence or absence of the GABA_A_R inhibitor. Phosphorylation levels of 4E-BP1 and S6K were analyzed by Western blotting 6 h post-GABA treatment. (**E and F**) P62 knockdown strengthens the antiviral effect of GABA, and this effect is abolished by GABA_A_R inhibition. After P62 silencing, shrimp were treated with GABA (2 μg) in the presence or absence of the GABA_A_R inhibitor and then infected with WSSV (5 × 10^5^ copies). WSSV load (**E**) and VP28 levels (**F**) were measured 48 h post-infection by qPCR and Western blotting, respectively. (**G**) P62 knockdown reduces shrimp survival upon GABA treatment. Shrimp were injected with siP62 or control siRNA. After P62 silencing, shrimp were administered GABA (2 μg) in the presence or absence of rapamycin. Survival rates were monitored every 12 h for 96 h (*n* = 30). All bar chart data are presented as mean ± SD of three or four replicates. ***, *P* < 0.001; **, 0.001 < *P* < 0.01; ns, no significant difference, as determined using Student’s *t*-test. The survival data are representative of three biological replicates and analyzed by log-rank (Mantel-Cox) test. The Western blotting images are representative of three independent replicates.

Excessive inhibition of autophagy can cause autophagosome accumulation and thereby impair cellular function (24–27). The autophagy-related protein P62 accumulates when autophagic activity is reduced (28–30). In addition, previous studies in mammals have shown that P62 can modulate GABA_A_R membrane localization (31, 32). Based on these observations, we hypothesized that GABA-induced P62 accumulation may act as a negative-feedback mechanism that restrains autophagy suppression and limits the detrimental effects of excessive autophagy inhibition in shrimp. To determine whether P62 is involved in GABA-mediated regulation of autophagy, we first measured P62 protein levels after GABA treatment. GABA treatment significantly increased P62 protein levels in shrimp (Fig. 6A). We next knocked down P62 by siRNA (Fig. 6B) and examined its effect on GABA signaling. P62 knockdown enhanced GABA-induced Ca²^+^ elevation in shrimp hemocytes (Fig. 6C). Consistently, P62 knockdown further enhanced GABA-induced phosphorylation of 4E-BP1 and S6K and strengthened the antiviral effect of GABA, and these effects were abolished by GABA_A_R inhibition (Fig. 6D through F). These results suggest that this negative-feedback mechanism limits the detrimental effects of excessive autophagy suppression in shrimp. Under P62-deficient conditions, 2 μg GABA reduced shrimp survival by approximately 30% within 96 h, whereas the same dose had no detectable effect in control shrimp (Fig. 6G). Importantly, rapamycin administration restored the survival of P62-deficient shrimp to a level comparable to that of the control group. Together, these findings indicate that P62 restrains GABA signaling through negative feedback and thereby limits the detrimental effects of excessive autophagy suppression.

## DISCUSSION

In this study, we show that a host signaling pathway restricts WSSV infection by suppressing virus-induced autophagy in shrimp. Our data show that GABA acts through a GABA_A_R-Ca^2+^-mTORC1 axis to inhibit autophagy, thereby reducing the early stage of WSSV infection and limiting infection. This response was further restrained by a P62-dependent feedback mechanism, which limited excessive autophagy suppression and reduced its detrimental effects. Together, these findings identify a host signaling mechanism that restricts WSSV infection by constraining virus-induced autophagy in shrimp.

Although autophagy can function as a cell-autonomous defense mechanism, many viruses have evolved strategies to exploit autophagy or autophagy-related processes to support entry, replication, assembly, or metabolic adaptation (33–35). Our previous work showed that WSSV hijacks lipid autophagy to mobilize triglycerides (TGs), increase free fatty acid availability, and support ATP production, thereby promoting viral entry in shrimp (6). The present study extends this model by showing that host signaling can antagonize this proviral autophagy program. GABA suppressed WSSV-induced autophagy and reversed the associated metabolic changes, ultimately inhibiting the early stage of WSSV infection. This interpretation is further supported by the non-additive effects of GABA and 3-MA, which suggest that GABA acts on the autophagy-dependent process exploited by WSSV rather than through a separate pathway. Together, these findings indicate that GABA limits WSSV infection by suppressing a proviral autophagy program that supports the early stage of WSSV infection.

Our data show that GABA suppresses virus-induced autophagy through a GABA_A_R-Ca^2+^-mTORC1 signaling pathway. GABA exerts its biological functions primarily through two receptor classes, the ionotropic GABA type A receptor and the metabotropic GABA type B receptor, which differ markedly in signaling mode (22). In shrimp, inhibition of GABA_A_R, but not GABA_B_R, abolished the effects of GABA on intracellular Ca^2+^ elevation, mTORC1 activation, autophagy suppression, and the early stage of WSSV infection, indicating that GABA_A_R is the receptor class required for this antiviral response. This receptor specificity fits with the rapid increase in intracellular Ca^2+^ after GABA treatment because GABAAR mediates fast ion flux across the plasma membrane (36). Calcium signaling has long been implicated in the control of autophagy, but its effects are clearly context dependent (37–39). In many settings, Ca^2+^ promotes autophagy through CaMKKβ-AMPK or PKC-related signaling (40–43). In other settings, elevated cytosolic or organelle-derived Ca^2+^ can suppress autophagy, including through calpain- or mTORC1-associated pathways (44–46). Our results place extracellular Ca^2+^ influx upstream of mTORC1 activation in the GABA-mediated antiviral pathway because blocking Ca^2+^ entry abolished GABA-induced phosphorylation of 4E-BP1 and S6K, restored autophagic activity, and eliminated the inhibitory effects of GABA on the early stage of WSSV infection, replication, and viral gene expression. Together, these findings support a model in which GABA_A_R-dependent Ca^2+^ influx promotes mTORC1 activation and thereby suppresses virus-induced autophagy in shrimp hemocytes.

A further major finding is that a feedback mechanism restrains GABA-induced autophagy suppression. Autophagy is essential for protein quality control, organelle turnover, and metabolic homeostasis, and both excessive autophagic activity and insufficient autophagic activity can be detrimental to cell and organismal viability (47–50). In the present study, GABA treatment increased P62 accumulation, and P62 knockdown enhanced GABA-induced Ca^2+^ influx, mTORC1 activation, and antiviral efficacy. More importantly, P62 deficiency rendered shrimp vulnerable to the detrimental effect of GABA treatment on survival, even though the same dose of GABA did not affect survival in control animals. These data indicate that P62 is not merely a passive marker of reduced autophagic activity, but an active feedback component that limits pathway strength and preserves host viability. Given previous reports in mammalian neurons that P62 can influence GABA_A_R membrane localization (31, 32), our results support a model in which GABA-induced autophagy suppression leads to P62 accumulation, which in turn dampens GABA signaling and prevents excessive inhibition of a process that remains necessary for host homeostasis. Thus, host suppression of the autophagy program exploited by WSSV appears to be constrained by a feedback mechanism that protects against the detrimental consequences of excessive autophagy inhibition.

**Fig 7:**
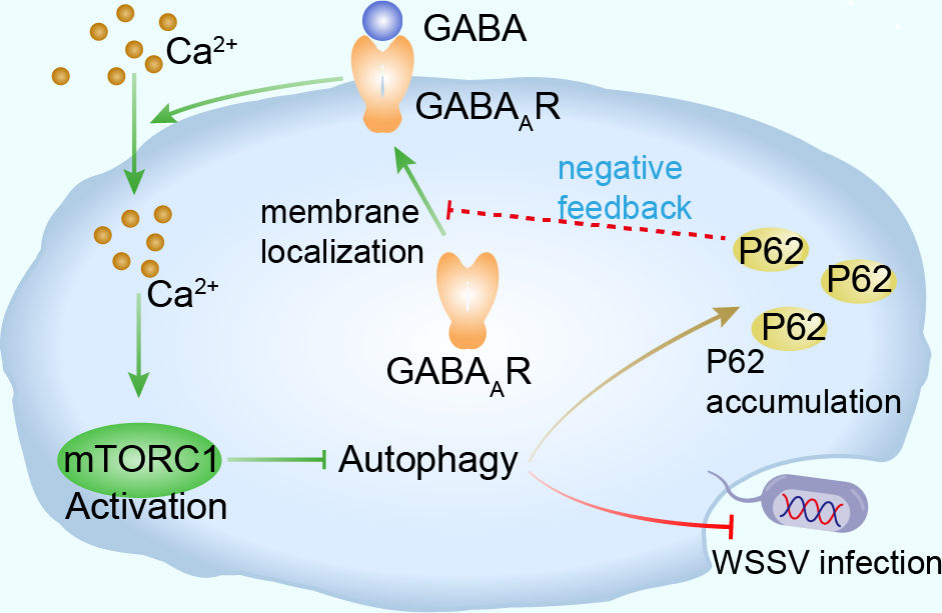
Model of the mechanism revealed in this study. GABA triggers GABA_A_R-dependent calcium influx, leading to mTORC1 activation. This signaling cascade suppresses WSSV infection by inhibiting autophagy. Reduced autophagic activity also causes P62 accumulation, which reduces GABA_A_R membrane localization and thereby attenuates GABA-mediated autophagy suppression. This feedback loop prevents excessive autophagy suppression and protects shrimp from the detrimental effects associated with abnormally low autophagic activity.

In summary, this study identifies a host signaling pathway that counteracts WSSV infection by suppressing virus-induced autophagy. GABA acts through a GABA_A_R- Ca^2+^-mTORC1 axis to inhibit a proviral autophagy program that supports the early stage of WSSV infection, and this pathway is further constrained by P62-dependent feedback that reduces the survival cost of autophagy suppression (Fig. 7). These findings show that neurotransmitter signaling can directly contribute to antiviral control in an invertebrate host and add a new layer to host regulation of WSSV infection.

## MATERIALS AND METHODS

### Experimental animals

Healthy kuruma shrimp (*Marsupenaeus japonicus*) with an average weight of 5 g were obtained from an aquaculture facility in Jimo City, Shandong, China. The shrimp were maintained in aerated seawater at 23°C and provided commercial feed daily. Shrimp were randomly allocated for experimental investigations.

### Virus inoculum

The WSSV inoculum used in this scientific research was kindly provided by the East China Sea Fisheries Research Institute in Shanghai, China. It was used to infect kuruma shrimp for virus propagation. The gills of dying shrimp were collected and homogenized in phosphate-buffered saline (PBS) (140 mM NaCl, 2.7 mM KCl, 10 mM Na_2_HPO_4_, 1.8 mM KH_2_PO_4_, pH 7.4) at a ratio of 1:10 (weight/volume) in an ice bath. The homogenate was freeze-thawed twice and then centrifuged at 3,000 × *g* for 10 min at 4°C. The supernatant was successively filtered through a 500-mesh sieve and a 0.45-μm filter, and the filtrate was collected. The virus titer was determined by the method described below. After determining the virus titer, the virus inoculum was stored at −80°C for later use. Before inoculation, the filtrate was diluted to an appropriate concentration with PBS as the virus inoculum. In this study, for virus infection assays, each shrimp was intramuscularly injected with a dose of 5 × 10⁵ (30 μL PBS) virus copies, and each shrimp was injected with an equal volume of PBS as a control.

The DIV1 inoculum used in this scientific research was a gift from the Third Institute of Oceanography, Ministry of Natural Resources in Xiamen, China. The cephalothorax (excluding the carapace) of shrimp infected with DIV1 was homogenized in PBS at a ratio of 1:20 (wt/vol) in an ice bath. The homogenate was centrifuged at 10,000 × *g* for 10 min at 4°C. The supernatant was sequentially filtered through a 500-mesh sieve and a 0.45 μm filter, and the filtrate was collected. Samples of the filtrate were tested to confirm the absence of other pathogenic microorganisms. The virus titer was determined as described below. After determining the virus titer, the inoculum was stored at −80°C. Before inoculation, the filtrate was diluted to an appropriate concentration with PBS as the virus inoculum. In this study, for virus infection assays, each shrimp was intramuscularly injected with a dose of 5 × 10^5^ (30 μL PBS) virus copies, and each shrimp was injected with an equal volume of PBS as a control.

### Determination of virus titer

The gene fragments of the structural protein 28 (*vp28*) of WSSV and the major capsid protein (*mcp*) of DIV1 were cloned into the pBlueScript vector. The recombinant plasmids were amplified in *Escherichia coli* DH5α. The concentration of the plasmids was measured using a Nanodrop ND 2000 spectrophotometer (Thermo Fisher Scientific, Waltham, MA, USA). The plasmid samples with calculated copy numbers were serially diluted.

Genomic DNA was extracted from the virus inoculum (100 μL) or the gills of virus-infected shrimp (10 mg) using the MagExtractor genomic DNA purification kit (Toyobo, Osaka, Japan, NPK-101).

The genomic DNA and the serially diluted plasmid samples were analyzed by quantitative PCR (qPCR). qPCR was performed using a CFX96 real-time system (Bio-Rad, Hercules, CA, USA) and iQ SYBR Green Supermix (Bio-Rad, 170-8882). The cycling conditions of qPCR were as follows: 94°C for 3 min, followed by 40 cycles (94℃ for 10 s, 60°C for 1 min), and finally a dissociation program from 65°C to 95°C. A standard curve was generated using the data of the plasmid samples and was used to quantify the virus copy number in the inoculum or gill tissue.

### RNAi

Small interfering RNAs corresponding to the gene sequences of shrimp *mTOR* and *P62* were synthesized to knock down the expression of specific genes. The scrambled siRNA corresponding to each siRNA was synthesized simultaneously as a control.

Appropriate small interfering RNA sequences were predicted using an online tool (biodev.extra.cea.fr/DSIR/DSIR). A pair of reverse complementary oligonucleotide sequences containing the T7 promoter and the small interfering RNA sequence was commercially synthesized. The siRNA was synthesized and purified using the T7 RNAi Transcription Kit (Vazyme, Nanjing, China; TR102) according to the manufacturer’s instructions. The siRNA was injected into shrimp with a dose of 30 μg per shrimp. Twenty-four hours after siRNA injection, qPCR was used to assess the RNAi efficiency with β*-actin* as the internal reference. The siRNAs and qPCR primers are listed in Table 1.

**TABLE 1:** Primers used in this study

Primers	Sequence (5′–3′)
RT-PCR	
vp28RTF	AGCTCCAACACCTCCTCCTTCA
vp28RTR	TTACTCGGTCTCAGTGCCAGA
mcpRTF	CGAGCACGTATGGGATGTGT
mcpRTR	CGCTCTGATCTGGGTCGAAA
ATG3RTF	CAAGTTCAAAGAGACGGGA
ATG3RTR	TCTGGAGGGAGATATGGTT
ATG5RTF	GGTGGCTCTGCTCTTCCT
ATG5RTR	GTGCTTCAAACCATCTGC
ATG10RTF	AAGGTGACGCCCAAAAAG
ATG10RTR	TTCCGTAATGCCAAGAGC
ATG12RTF	ATAAACCTGCGACTTCCC
ATG12RTR	GCTTCTCCCCTTCCACTG
GABA_A_RRTF	TACCTTGGCACCTGTTTCG
GABA_A_RRTR	CACTGTCTGCTTCTGGGGAT
mTORRTF	CTCCTCACGGTCTGGTTTG
mTORRTR	CGGACGACTTTGCTGCTAC
P62RTF	AGCACTCCGAACACCGTA
P62RTR	TCCACCTCCAAATGAAGC
β-actinRTF	CAGCCTTCCTTCCTGGGTATGG
β-actinRTR	GAGGGAGCGAGGGCAGTGATT
RNAi	
mTOROligo-1	GATCACTAATACGACTCACTATAGGGGTGCAGGCAGATCCTTGAGAATT
mTOROligo-2	AATTCTCAAGGATCTGCCTGCACCCCTATAGTGAGTCGTATTAGTGATC
mTOROligo-3	GATCACTAATACGACTCACTATAGGGTTCTCAAGGATCTGCCTGCATT
mTOROligo-4	AATGCAGGCAGATCCTTGAGAACCCTATAGTGAGTCGTATTAGTGATC
Scramble-mTOROligo-1	GATCACTAATACGACTCACTATAGGGGACTGGGTACCGCATAAGTGTT
Scramble-mTOROligo-2	AACACTTATGCGGTACCCAGTCCCCTATAGTGAGTCGTATTAGTGATC
Scramble-mTOROligo-3	GATCACTAATACGACTCACTATAGGGGCACTTATGCGGTACCCAGTCTT
Scramble-mTOROligo-4	AAGACTGGGTACCGCATAAGTGCCCCTATAGTGAGTCGTATTAGTGATC
GABA_A_ROligo-1	GATCACTAATACGACTCACTATAGGGCGACGACTCTGACGAAGATTT
GABA_A_ROligo-2	AAATCTTCGTCAGAGTCGTCGCCCTATAGTGAGTCGTATTAGTGATC
GABA_A_ROligo-3	GATCACTAATACGACTCACTATAGGGATCTTCGTCAGAGTCGTCGTT
GABA_A_ROligo-4	AACGACGACTCTGACGAAGATCCCTATAGTGAGTCGTATTAGTGATC
Scramble-GABA_A_ROligo-1	GATCACTAATACGACTCACTATAGGGGATCGACAGTACGACTCGATT
Scramble-GABA_A_ROligo-2	AATCGAGTCGTACTGTCGATCCCCTATAGTGAGTCGTATTAGTGATC
Scramble-GABA_A_ROligo-3	GATCACTAATACGACTCACTATAGGGTCGAGTCGTACTGTCGATCTT
Scramble-GABA_A_ROligo-4	AAGATCGACAGTACGACTCGACCCTATAGTGAGTCGTATTAGTGATC
P62Oligo-1	GATCACTAATACGACTCACTATAGGGGCATTGCCACAAACTTTACCTTT
P62Oligo-2	AAAGGTAAAGTTTGTGGCAATGCCCCTATAGTGAGTCGTATTAGTGATC
P62Oligo-3	GATCACTAATACGACTCACTATAGGGAGGTAAAGTTTGTGGCAATGCTT
P62Oligo-4	AAGCATTGCCACAAACTTTACCTCCCTATAGTGAGTCGTATTAGTGATC
Scramble-P62Oligo-1	GATCACTAATACGACTCACTATAGGGGACATATCCTCACGTCTCATT
Scramble-P62Oligo-2	AATGAGACGTGAGGATATGTCCCCTATAGTGAGTCGTATTAGTGATC
Scramble-P62Oligo-3	GATCACTAATACGACTCACTATAGGGTGAGACGTGAGGATATGTCTT
Scramble-P62Oligo-4	AAGACATATCCTCACGTCTCACCCTATAGTGAGTCGTATTAGTGATC
Recombinant expression	
vp28F	GCCATGGCTGATATCGGATCCATGGATCTTTCTTTCACTCTTTCGG
vp28R	GTGGTGGTGGTGGTGCTCGAGTTACTCGGTCTCAGTGCCAGAGT
P62F	GCCATGGCTGATATCGGATCCCCAAACCCACAGCAGGTTGC
P62R	GTGGTGGTGGTGGTGCTCGAGAGGTTGTTGAGTTGTGGTCTGCG

### Gene expression analysis

The expression of *vp28*, *mcp*, *ATG3*, *ATG5*, *ATG10*, *ATG12*, *mTOR*, and *P62* was analyzed by qPCR, and the method was similar to the above. Meanwhile, the expression of β*-actin* was detected as an internal reference. The PCR data were processed using the 2^−∆∆CT^ method. The expression level was presented relative to the control sample (the first column of the gene expression histogram).

### Western blotting analysis

Western blotting analysis was used to analyze the level changes of P62, GABARAP proteins GABARAPL1 and GABARAPL2 of shrimp, and the structural protein 28 (VP28) of white spot syndrome virus, as well as the phosphorylation levels of p70 ribosomal S6 kinase (S6K) and eukaryotic translation initiation factor 4E-binding protein 1 (4E-BP1). The β-actin was used as an internal reference.

Shrimp hemocytes were collected using a precooled anticoagulant (0.67 M NaCl, 33 mM C_6_H_5_Na_3_O_7_, and 9 mM EDTA) and then resuspended in RIPA (Beyotime, Nanjing, China; P0013D) to extract total protein. The suspension was placed on ice and incubated for 10 min. It was then centrifuged at 12,000 × *g* for 10 min at 4°C to obtain the supernatant containing the protein sample. The protein sample was mixed with SDS-PAGE loading buffer (Beyotime, P0015) and heated in a 100°C metal bath for 10 min. Next, 100 μg of protein per lane was separated by 12.5% SDS-PAGE and transferred to a nitrocellulose membrane using a Jim-X semidry transfer apparatus (Jim-X, Dalian, China). The membrane was blocked with 3% skim milk for 1 h before incubation with the appropriate primary antibody for 4 h. After three TBS (150 mM NaCl, 25 mM Tris-HCl, pH 7.6) washes, the membrane was incubated with the HRP-conjugated secondary antibody for 2 h, concluding with three TBS washes. The immunoreactive protein signals were visualized with High-sig ECL Western blotting substrate (Tanon, Shanghai, China; 180-5001) and captured using the Tanon 5200 chemiluminescence imaging system (Tanon).

The antibodies against β-actin, VP28, and P62 were prepared by immunizing rabbits with recombinant proteins in the laboratory, and the antibody dilution ratio was 1:500.

Rabbit antihuman GABARAP monoclonal antibody (Abcam, Cambridge, UK; ab109364), rabbit antihuman 4E-BP1 phosphorylated polyclonal antibody (CST, Danvers, USA; 2855), and rabbit antihuman S6K phosphorylated polyclonal antibody (ABclonal, Wuhan, China; AP0564) were used at an antibody dilution ratio of 1:2,000.

Horseradish enzyme-labeled goat anti-rabbit antibody (Zhongshan, Beijing, China; ZB-2301) was used at an antibody dilution ratio of 1:3,000.

### Generation of recombinant proteins

The gene fragments encoding the mature peptides of Mjp62, Mjβ-actin, and VP28 were cloned into pGEX-4T-2 and pET30a (+) plasmids, respectively, and then transformed into *E*. *coli* Transetta (DE3) strains. The primers used for amplification of these gene fragments are listed in Table 1. Recombinant protein expression was induced with 0.2 mM isopropyl-β-D-thiogalactoside at 28°C. The recombinant proteins were expressed in soluble form and purified using ProteinIso GST column material (TransGen Biotech, Beijing, China; DP-201) and ProteinIso Ni-NTA column material (TransGen Biotech, DP-101), respectively. Endotoxin was removed by washing with precooled 0.1% Triton X-114. Protein concentration was determined using the Bradford kit (Sangon, Shanghai, China; C503031). The purified soluble proteins were refolded by complete dialysis with gradient dialysate and stored at −80°C before use.

### Flow cytometry analysis

WSSV virus particles stained with FITC (Sigma-Aldrich, USA; 1.24546) (5 × 10^5^ copies) were injected into the hemocoel of shrimp. Two hours later, the hemolymph of five shrimp was collected and mixed with an equal volume of precooled anticoagulant. The mixture was centrifuged at 800 × *g* for 10 min at 4°C, and the supernatant was discarded to collect hemocytes. The hemocytes were resuspended in precooled artificial seawater (436 mM NaCl, 8 mM Na_2_HPO_4_, 10 mM KCl, 5 mM KH_2_PO_4_) and filtered through a 300-mesh cell sieve. Flow cytometry was performed using an ImageStreamX Mark II flow cytometer (Merck, Kenilworth, NJ, USA) to detect FITC fluorescence intensity in individual cells. A total of 10,000 cells were analyzed. The data were processed with IDEAS software (Merck).

### Immunofluorescence experiment

Two hours after GABA administration, FITC-labeled WSSV particles were injected into the hemocoel of shrimp. Two hours later, the hemolymph was collected into precooled anticoagulant containing 4% paraformaldehyde, incubated for fixation, and centrifuged at 800 × *g* for 10 min at 4°C to collect hemocytes. The hemocytes were resuspended in artificial seawater, and the suspension was smeared onto polylysine-coated slides. Then the slides were washed three times with PBS. Cells were incubated with 4′,6-diamidino-2-phenylindole (AnaSpec, San Jose, CA, USA; AS-83210) for 10 min in the dark at room temperature. Following three PBS washes, the slides were imaged on an LSM 900 confocal microscope (Zeiss, Oberkochen, Germany). Virus-positive hemocyte percentages were determined by cell counting. Four fields of view (≥100 cells per field) were analyzed. Images were processed with ZEN software (Zeiss). Control shrimp were injected with PBS, followed 2 h later by FITC-labeled WSSV injection.

### Survival rate experiment

To confirm the effect of GABA administration on the survival of shrimp, administration gradients of 0, 1, 2, and 5 μg were set, with 30 shrimp in each group. The survival rate of shrimp in each group was recorded every 12 h.

To confirm whether GABA administration can reduce the death of shrimp caused by WSSV infection, WSSV inoculum (1 × 10⁶ copies) was injected 2 h after GABA administration. The control group of shrimp was injected with only WSSV inoculum. The survival rate of shrimp in the two groups was recorded every 12 h. The survival rate data of shrimp were analyzed using the Mantel-Cox test in GraphPad Prism 8.

### Determination of triglyceride, free fatty acid, and ATP levels

Two hours after GABA administration, WSSV inoculum (1 × 10⁶ copies) was injected into the hemocoel of shrimp. Twelve hours later, the hemolymph of five shrimp was collected into an equal volume of precooled anticoagulant. The mixture was centrifuged at 800 × *g* for 10 min at 4°C, and the supernatant was discarded to obtain hemocytes. The hemocytes of shrimp were resuspended with precooled artificial seawater and divided into three equal parts. The TG level was detected using a TG content detection kit (Solarbio, Beijing, China; BC0620); the FFA level was detected using a free fatty acid content detection kit (Solarbio, BC0590); and the ATP level was detected using an ATP level detection kit (Beyotime, Wuhan, China; S0027) according to the manufacturer’s instructions.

### Intracellular Ca^2+^ concentration detection

The hemolymph of five shrimp was collected and mixed with an equal volume of precooled anticoagulant. The mixture was centrifuged at 800 × *g* for 10 min at 4°C, and the supernatant was discarded to obtain hemocytes. The hemocytes were washed once with precooled artificial seawater. The hemocytes were resuspended in L15 medium, transferred to cell culture dishes, and cultured overnight at 27°C. The medium was removed, and hemocytes were incubated with artificial seawater containing Fluo 3-AM (Solarbio, F8840) (final concentration 3 μM) for 30 min at 27°C. After washing with artificial seawater, hemocytes were immersed in artificial seawater and placed under a confocal microscope. Fluorescence at 527 nm was captured every 6 s for 1 min. GABA was then added, and imaging continued for 2 min. Calcium chloride (final concentration 1 mM) was subsequently added, and imaging proceeded for 4 min. Fluorescence intensity of each image was acquired using ImageJ software (NIH, Bethesda, MD, USA), followed by statistical analysis. The relative fluorescence values were expressed as ratios to the initial data.

### Chemicals

GABA (A600040) was purchased from Sangon. RAPA (S1039) was dissolved in PBS containing 10% DMSO, with a dosage of 20 μg per shrimp. 3-MA (S2767), calcium ion channel blocker (SKF96365, S7999), GABA_A_R inhibitor (SR95531, E1247), and GABA_B_R inhibitor (CGP52432, S0303) were dissolved in PBS, with dosages of 5, 5, 5, and 2 μg per shrimp, respectively. Their solvents served as control treatments; all reagents were purchased from Selleck Chemicals (Houston, TX, USA).

### Statistical analysis

The data in histograms (virus load, gene expression, triglyceride, free fatty acid, and ATP content determination) are expressed as mean ± SD from three or four independent replicates. Immunocytochemistry, blot, fluorescence microscopy, and flow cytometry images are representative of three independent experiments. A minimum of five shrimp were used per sample preparation. Statistical significance was assessed by *t*-test or one-way ANOVA, with analysis performed using GraphPad Prism (GraphPad Inc., La Jolla, CA, USA).

## Data Availability

All data are included in this article. Additional information is available from the corresponding author.
